# Effect of remimazolam vs propofol in high-risk patients undergoing upper gastrointestinal endoscopy: a non-inferiority randomized controlled trial

**DOI:** 10.1186/s13063-024-07934-z

**Published:** 2024-01-27

**Authors:** Zhi Li, Daming Yuan, Yu Yu, Jie Xu, Weili Yang, Li Chen, Nanbo Luo

**Affiliations:** 1 Department of Anesthesiology, The Second People’s Hospital of Futian District Shenzhen, No. 27 Zhong Kang Road, Futian District, Shenzhen, 518000 China; 2 Department of Gastroenterology, The Second People’s Hospital of Futian District Shenzhen, No. 27 Zhong Kang Road, Futian District, Shenzhen, 518000 China; 3 Department of Anesthesiology, Inst Translat Med, Shenzhen Second People’s Hospital/The First Affiliated Hospital of Shenzhen University, Shenzhen, 518000 China

**Keywords:** Procedural sedation, Upper gastrointestinal endoscopy, High-risk patients, Remimazolam, Randomized controlled trial

## Abstract

**Background:**

Procedural sedation is essential for optimizing upper gastrointestinal endoscopy, particularly in high-risk patients with multiple underlying diseases. Respiratory and circulatory complications present significant challenges for procedural sedation in this population. This non-inferiority randomized controlled trial aims to investigate the safety and comfort of remimazolam compared to propofol for procedural sedation during upper gastrointestinal endoscopy in high-risk patients.

**Methods:**

A total of 576 high-risk patients scheduled to undergo upper gastrointestinal endoscopy are planned to be enrolled in this study and randomly allocated to either the remimazolam or propofol group. The primary outcome measure is a composite endpoint, which includes (1) achieving a Modified Observer’s Alertness/Sedation scale (MOAA/S) score ≤ 3 before endoscope insertion, (2) successful completion of the endoscopic procedure, (3) the absence of significant respiratory instability during the endoscopy and treatment, and (4) the absence of significant circulatory instability during the examination. The noninferiority margin was 10%. Any adverse events (AEs) that occur will be reported.

**Discussion:**

This trial aims to determine whether remimazolam is non-inferior to propofol for procedural sedation during upper gastrointestinal endoscopy in high-risk patients, regarding success rate, complication incidence, patient comfort, and satisfaction.

**Trial registration {2a and 2b}:**

Chinese Clinical Trial Registry ClinicalTrials.gov ChiCTR2200066527. Registered on 7 December 2022.

**Supplementary Information:**

The online version contains supplementary material available at 10.1186/s13063-024-07934-z.

## Background {6a and 6b}

Gastrointestinal endoscopy is widely used for the diagnosis and treatment of digestive diseases. Midazolam and propofol are commonly utilized for procedural sedation during upper gastrointestinal endoscopy procedures [1]. Although both propofol and midazolam have demonstrated efficacy, they present specific limitations. Propofol provides desirable sedation depth and a short half-life (t1/2), enabling rapid patient recovery. However, it may cause respiratory depression, hypoxemia, and hypotension, necessitating continuous vital sign monitoring, including respiration. Midazolam, widely used, allows for easier sedation level titration compared to propofol, requiring less intensive monitoring. Its drawback is a half-life of 1–3 h [2, 3]. Remimazolam, a novel sedative and ester-based benzodiazepine, is rapidly hydrolyzed by liver esterase into its inactive carboxylic acid metabolite. With a shorter half-life than midazolam, remimazolam exhibits improved safety, facilitating swift awakening and early cognitive function restoration [4]. Phase III clinical trials of remimazolam for upper gastrointestinal endoscopy have demonstrated sedative effects and safety profiles in ASA grade (Appendix 1) I–II patients that are not inferior to propofol, with the sedative regimen outperforming propofol in certain aspects [5]. Procedural sedation safety and efficacy are primarily influenced by the patient’s physical condition. As ASA levels rise, the risk of adverse events during endoscopy increases [6]. Considering the requirements of upper gastrointestinal endoscopy, a safe and comfortable diagnostic and treatment experience during upper gastrointestinal endoscopy for these patients is important.

### Objectives {7}

This study aims to investigate high-risk patients (ASA levels III and IV) to ascertain if remimazolam can ensure a safe and comfortable diagnostic and treatment experience during upper gastrointestinal endoscopy for these patients. Additionally, this study seeks to evaluate whether remimazolam can provide optimal examination conditions for endoscopic practitioners, thereby laying a solid foundation for the widespread implementation of remimazolam in procedural sedation.

## Methods/design

### Trial design {8}

**Fig. 1:**
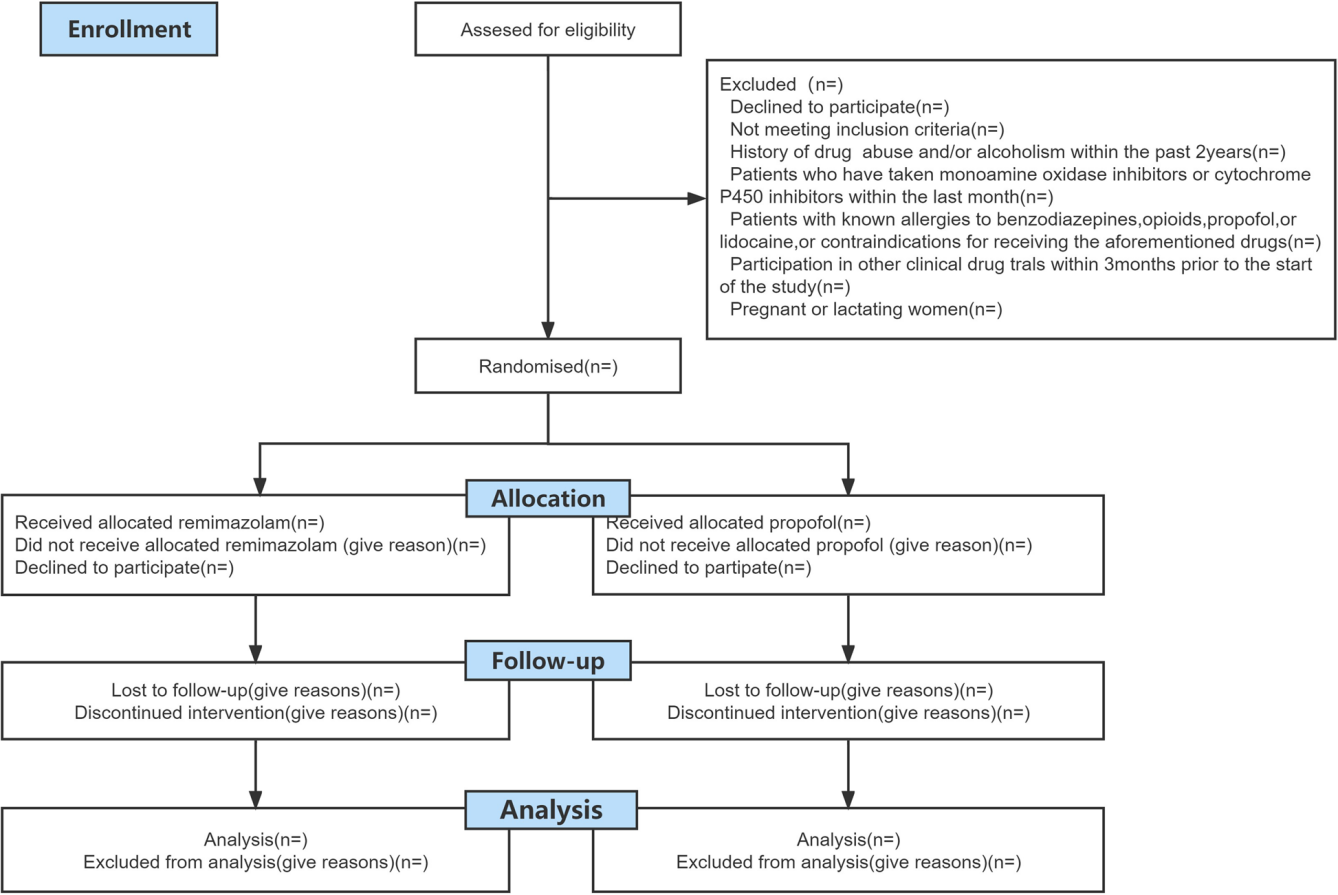
Trial Flow

We have developed a single-center, randomized, parallel group controlled, non-inferiority trial to compare the sedative effects of remimazolam and propofol in high-risk populations undergoing upper gastrointestinal endoscopy. A total of 576 high-risk patients scheduled for upper gastrointestinal endoscopy will be enrolled and randomly allocated to either the remimazolam group or the propofol group using a block randomization method (Fig. 1). This study adheres to the principles of Good Clinical Practice for Drug Trials, in accordance with the Declaration of Helsinki. The SPIRIT checklist is provided in Additional file 1.

### Study setting {9}

The study will be conducted in the Second People’s Hospital of Futian District, Shenzhen, China.

#### Inclusion criteria {10}

Participants will be enrolled with the following criteria in this study: (1) age ≥ 18 years old, (2) patients scheduled to undergo upper gastrointestinal endoscopy or treatment under sedation, (3) the American Society of Anesthesiologists (ASA) classification of grade III or grade IV, and (4) patients who provide informed consent to participate in the study.

#### Exclusion criteria

Subjects will be excluded if they meet one of the following criteria: (1) Patients who decline sedation and anesthesia; (2) history of drug abuse and/or alcoholism within the past 2 years; (3) patients who have taken monoamine oxidase inhibitors or cytochrome P450 inhibitors within the last month; (4) patients with known allergies to benzodiazepines, opioids, propofol, or lidocaine, or contraindications for receiving the aforementioned drugs; (5) participation in other clinical drug trials within 3 months prior to the start of the study; (6) pregnant or lactating women.

#### Withdrawal criteria

For patients who do not meet the criteria, detailed reasons will be documented, and a case report form (CRF) will be retained for reference. The outcomes of these cases will be excluded from subsequent analyses of treatment effectiveness: (1) Loss of subject follow-up, (2) subject refusal to continue follow-up, (3) subject or their legally authorized representative requests withdrawal from the study, (4) investigator determines that the subject is unsuitable for continued study participation, (5) inability to implement trial interventions, and (6) test data not recorded.

### Randomization and allocation concealment {16}

The randomization method employs variable block randomization with random-sized blocks. Professional statisticians not involved in data management utilize the Data Web data collection management system to generate a central random sequence, randomly allocating subjects in a 1:1 ratio and dividing them into two groups. Stratification is based on whether upper gastrointestinal endoscopic invasive treatment is planned, as this factor may influence the duration of the procedure and potentially affect the sedation success rate. Random numbers are placed in coded, opaque envelopes and entrusted to personnel not involved in anesthesia or data collection for management.

### Blinding

#### Who will be blinded {17a}

The intervention drugs in this study, propofol and remimazolam, have distinct properties and dosages, precluding the possibility of blinding the interventionist. However, the drug administration process will be concealed from the subject during drug injection, implementing a single-blind design in which only the patient is blinded. The study data collector remains mutually inaccessible. The outcome assessors in the study are the interventionists, who are not blinded to the study. However, the data analysis is conducted by individuals who did not participate in the intervention measures and are blinded to the participants’ identities.

####  Procedure for unblinding if needed{17b}

In the context of clinical research, the act of unblinding may be deemed justifiable under certain circumstances, including the following:

1. Emergencies or adverse events: Unblinding may be warranted when a participant encounters a severe adverse event or medical emergency. In such instances, awareness of the specific intervention received becomes essential for informed and appropriate medical management.
2. Completion of the study: Upon the culmination of the study, participants are frequently apprised of the particulars regarding the nature of the intervention they were assigned. This post-study disclosure is a customary practice designed to ensure transparency and provide participants with comprehensive insights into the conducted research.
3. Unblinding requests: In the event of a participant’s request to know their intervention assignment, the research team will disclose this information following the trial procedures and ethical considerations.

### Interventions {11a}

**Fig. 2:**
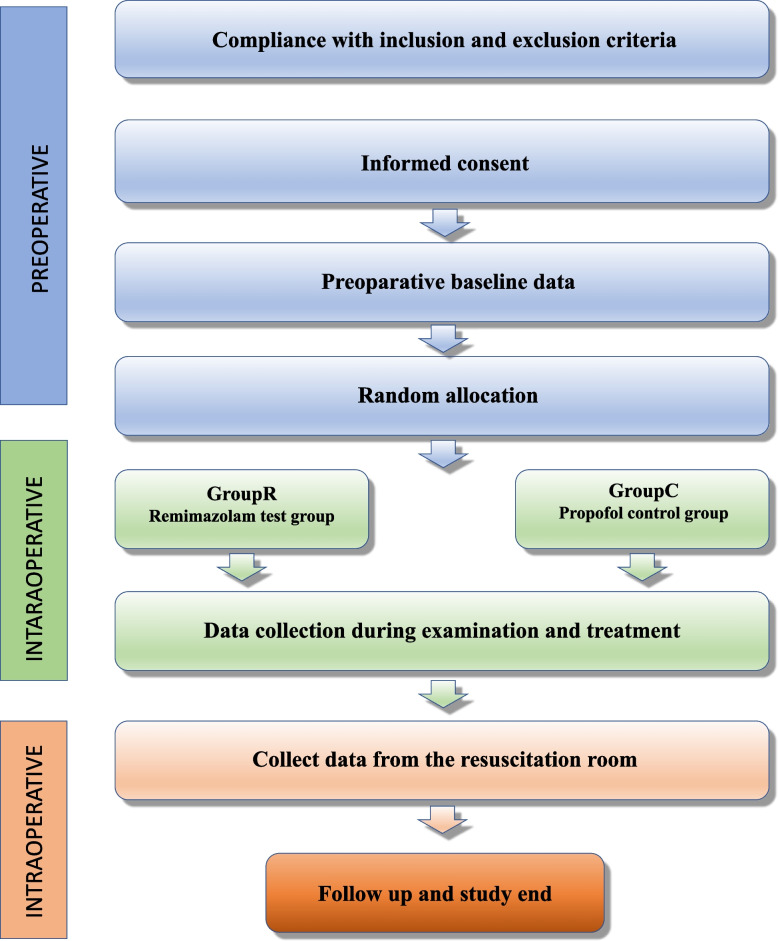
Technical RoadmapA

**Fig. 3:**
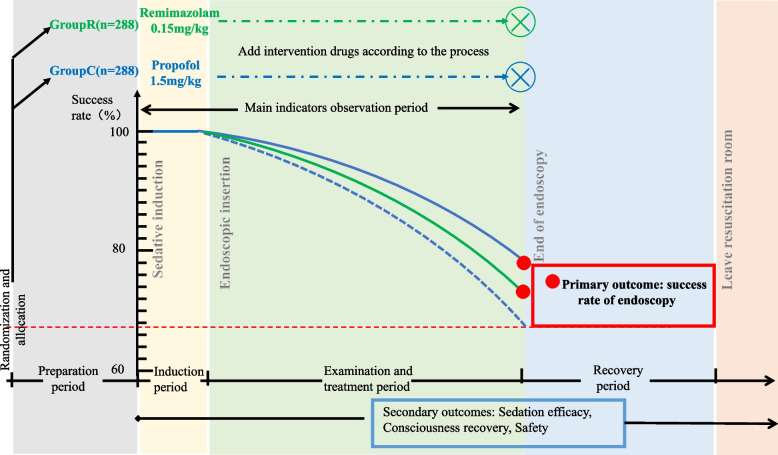
Technical RoadmapB

Upon obtaining signed informed consent from the subject, the following process is executed (Figs. 2 and 3):

1. Screening period
  After the patient enters the endoscopy examination room and decides to undergo upper gastrointestinal endoscopy treatment, the researcher collects information on the patient’s demographics, medical condition, and proposed examination plan. An independent anesthesiologist evaluates the patient’s examination plan before randomization, and eligible patients are selected based on the inclusion/exclusion criteria. Patients should be assessed by researchers who are knowledgeable about the protocol and authorized to do so in a quiet environment. The study’s purpose, methods, potential benefits, and risks must be thoroughly explained to the patient and/or their representative, and data collection and follow-up procedures before, during, and after the examination must be detailed.
  The researchers should address the patient and their family members’ questions in detail, allowing sufficient time for consideration and obtaining informed consent from the patient and/or their representative on a completely voluntary basis. The assessor must not be involved in the patient’s examination, sedation, or anesthesia management plan.
2. *Sedation management plan*: An anesthetist with the title of attending physician or above will implement the sedation and anesthesia plan. The patient enters the endoscopy consultation room’s preparation area and takes 10 g of lidocaine gel (containing 0.2 g of lidocaine) orally 5 min before the examination. The patient assumes a left lateral position on the treatment bed in the endoscopy consultation room, and basic vital signs including noninvasive blood pressure (NIBP) of the right upper arm, electrocardiogram, and pulse oxygen saturation (SpO_2_) are monitored. Oxygen is continuously administered through a 4L/min nasal cannula throughout the procedure until the patient fully awakens. A venous line is established, and sufentanil 5 μg is administered intravenously. Patients scheduled for upper gastrointestinal endoscopy are randomized to receive one of two sedative drug infusions: propofol in group C or remimazolam in group R. In group C, patients receive a single dose of propofol at 1.5 mg/kg, while in group R, patients receive a single dose of remimazolam at 0.15 mg/kg, both administered within 1 min. According to the domestic expert consensus, moderate or deep sedation is required for upper gastrointestinal endoscopic examination. Therefore, the MOAA/S score (Appendix 2) should be assessed 1 min after propofol injection. If the MOAA/S score is greater than 3 points, propofol 0.5 mg/kg or remimazolam 0.05 mg/kg should be administered repeatedly until the MOAA/S score is less than or equal to 3 points, with a maximum of five repetitions. The VAS (Appendix 3) should be assessed during propofol or remimazolam injection.
3. *Intraoperative monitoring*: Intraoperative monitoring includes electrocardiogram, noninvasive blood pressure, pulse oxygen saturation, and, when necessary, body temperature, urine output, end-tidal carbon dioxide concentration, and blood glucose levels.
4. *Sedation maintenance plan*: During the examination procedure, the chief anesthesiologist determines whether to administer additional trial medication based on the surgical process and duration. Propofol (0.5 mg/kg) or remimazolam (0.05 mg/kg) is administered in single doses with a minimum interval of 1 min to maintain the patient at an appropriate level of sedation (MOAA/S score ≤ 3). If sedatives are insufficient to maintain appropriate sedation during the examination, the investigator may decide to use alternative sedative agents (midazolam).
5. *Examination plan*: Based on the type of lesion, the endoscopic examination of the upper digestive tract in our hospital may include, but is not limited to, upper gastrointestinal inflammation, tumors, ulcers, polyps, gastroesophageal varices, and gastrointestinal strictures. According to the type of endoscopic treatment of the upper gastrointestinal tract, it may include, but is not limited to, sclerotherapy, argon plasma coagulation, high-frequency electrocautery, and submucosal dissection. Various procedures are conducted following established clinical surgical protocols.
6. *Postoperative management plan*: After the examination or procedure, the patient enters the anesthesia recovery room, where continuous monitoring of ECG, noninvasive blood pressure, and oxygen saturation occurs. The MOAA/S score is assessed every 5 min. Once the score reaches 5 points for three consecutive assessments, and blood pressure, heart rate, and oxygen saturation are normal and stable, the patient can leave the recovery room accompanied by a companion. In case of special circumstances, refer to the adverse event handling procedures.

### Criteria for discontinuation or modification of allocated interventions{11b}

We do not plan to intentionally alter the specified interventions. However, it should be emphasized that participation in all the interventions detailed in the program is at the discretion of the individual participants. They have the freedom to choose whether to engage in or omit any of the intervention measures. Thus, if participants perceive any aspect of the intervention as less beneficial, they may opt out of that component.

The attending anesthesiologist has the authority to discontinue a participant’s involvement in the trial at any point for medical reasons. This responsibility, including the assessment of factors such as adverse reactions, lies solely with the attending anesthesiologist. Furthermore, participants have the right to withdraw from the study whenever they wish by revoking their consent.

### Strategies to improve adherence to interventions {11c}

- Comprehensive informed consent process: During the recruitment phase, ensure the provision of clear, detailed, and easily comprehensible informed consent documents to participants. This documentation should encompass information on research objectives, procedures, potential risks, and benefits. Guarantee that participants possess a thorough understanding and willingly consent to participate in the study.
- Collaborative efforts within the medical team: Ensure effective collaboration among members of the medical team to collectively address issues that may impact adherence. A unified medical team can adeptly tackle challenges related to participant compliance.
- Anonymity and privacy safeguards: Reassure participants about the protection of their data and strive to ensure anonymity whenever feasible. This approach serves to alleviate concerns and bolster participant trust in the research process.

These strategies aim to enhance participant understanding, foster collaboration within the medical team, and prioritize the safeguarding of participant data and privacy.

### Relevant concomitant care permitted or prohibited during the trial {11d}

In our study, the endoscopic examinations and treatments are conducted following standard care and interventions as per routine clinical practice. This approach ensures that all procedures align with the established norms of medical care for such procedures.

Additionally, it is important to note that any concomitant treatments or interventions that fall under the exclusion criteria of our trial are strictly prohibited. We have implemented this prohibition to maintain the integrity of the trial and to ensure that the outcomes are not influenced by external variables. This includes any interventions that directly result from or are related to the exclusion criteria.

### Data Collection {18a}

#### Preoperative data collection

The baseline data collection will be conducted when the subjects enter the gastroscopy treatment room and are in a calm state. Upon obtaining informed consent from the subjects, the preoperative interviewer shall complete the baseline data collection as follows:

- Demographic information, including name, gender, date of birth, height, and weight
- Preoperative diagnosis, including comorbidities (such as coronary heart disease, history of cerebrovascular accidents, hypertension, diabetes, chronic obstructive pulmonary disease, abnormal liver and kidney function), medication history (ACE inhibitors, ARBs, β-blockers, insulin, metformin, aspirin, clopidogrel, salbutamol, aminophylline, ipratropium bromide, etc.), smoking and smoking cessation history, and history of anesthesia and sedation.
- Severity of the disease or physical condition, ASA classification (refer to Appendix 1, with a requirement for participants to be classified as ASA III–IV).
- Baseline values of NIBP, HR, and SpO_2_ before the examination.
- Upper gastrointestinal endoscopy examination and planned examination location (esophagus and/or stomach).

#### Intraoperative data collection

Starting from the administration of intervention drugs, test the MOAA/S score every 30 s for 5 min, and then test every 2 min until the examination and operation are completed.

Record the time interval (seconds) from the start of intervention drugs to the time when MOAA/S score ≤ 3.

Record the presence of injection pain during the administration of intervention drugs using a visual analog scale (VAS) ranging from 0 to 10, where 0 indicates no pain and 10 indicates maximum pain (see Appendix 3). This assessment is conducted 1 min and 20 min after the start of drug administration or every 5 min thereafter if the patient remains sedated until the patient can complete the scale.

Record the MOAA/S score when the endoscope is smoothly placed in the esophagus.

Record the dose of intervention drugs used when the endoscope is smoothly inserted into the esophagus (including additional and rescue drugs, types, frequency, and dosage).

Measure NIBP every 3 min from the start of intervention drugs until the end of the examination.

Continuously monitor SpO_2_ until the end of the examination and record the minimum SpO_2_ during the procedure.

Record the total duration of the examination and operation (in minutes).

Record the types, frequency, and dosage of additional or rescue drugs during the examination, along with the corresponding time points.

Record the number of interruptions during the examination and the reasons for interruption, along with the time elapsed since drug administration began.

Record adverse events and their corresponding times during the period from drug administration to the end of the examination (see 1.10), such as respiratory spasms, reflux aspiration, coughing during endoscope entry into the oral cavity, SpO_2_ below 90% due to repeated coughing, SpO_2_ below 90% due to respiratory depression, positive pressure mask ventilation, jaw support-assisted ventilation, systolic blood pressure < 80 mmHg, or the need for vasopressors, systolic blood pressure ≥ 180 mmHg, *HR* ≤ 55 beats/min, *HR* > 100 beats/min, and the frequency and conditions of severe arrhythmias.

Operator satisfaction score (0–10 points, with 0 indicating very dissatisfied and 10 indicating very satisfied).

Post-examination diagnosis: Upper gastrointestinal inflammation, tumors, ulcers, polyps, esophagogastric varices, gastrointestinal stenosis, etc. and surgical procedures: endoscopic hemostasis (clipping, electrocoagulation, band ligation), sclerotherapy, argon plasma coagulation, high-frequency electrocautery, etc.

#### Recovery room data collection

Immediately after the examination, the patient is transported to the recovery room until fully awake.

After entering the recovery room, test the MOAA/S score every 5 min and record it.

Record the time required from the end of the operation to the patient’s MOAA/S score recovering to 5 points (need to reach 5 points continuously for three times, choose the first time with a score of 5).

Measure NIBP every 3 min after entering the recovery room and record it.

Continuously monitor SpO_2_ until the end of the examination and record the minimum SpO_2_ during the procedure.

Adverse events occurring in the recovery room (see 1.10), such as SpO_2_ below 90%, positive pressure mask ventilation, jaw support-assisted ventilation, systolic blood pressure < 80 mmHg or the need for vasopressors, systolic blood pressure ≥180 mmHg, *HR* ≤ 55 beats/min, and *HR* > 100 beats/min

When the subjects meet the discharge criteria, record their anesthesia satisfaction score (0–10 points, with 0 indicating very dissatisfied and 10 indicating very satisfied).

Whether flumazenil was used for antagonism in case of delayed awakening.

When the patient’s MOAA/S score recovers to 5 points, use the modified Brice questionnaire to determine if the patient was aware during the procedure.

Collect information on the operator’s years of experience.

#### Postoperative day 1 data collection

Collect pathological data on the first day after the operation, conduct telephone follow-up, and record relevant adverse events.

### Strategies for enhancing participant retention and ensuring comprehensive follow-up {18b}

There is a follow-up period, and it consists of a postoperative follow-up scheduled for one day. Participants will be assessed during this follow-up to monitor their progress and address any immediate concerns.

To promote participant retention and ensure complete follow-up, we have implemented comprehensive strategies. Typically, comprehensive follow-up is ensured by adhering to the recommendations of the anesthesiologist. These include regular communication with participants by telephone. Moreover, a meticulous effort is made to capture as many planned outcomes as possible for all participants, including those who discontinue their involvement in the study.

### Outcomes {12}

**Fig. 4:**
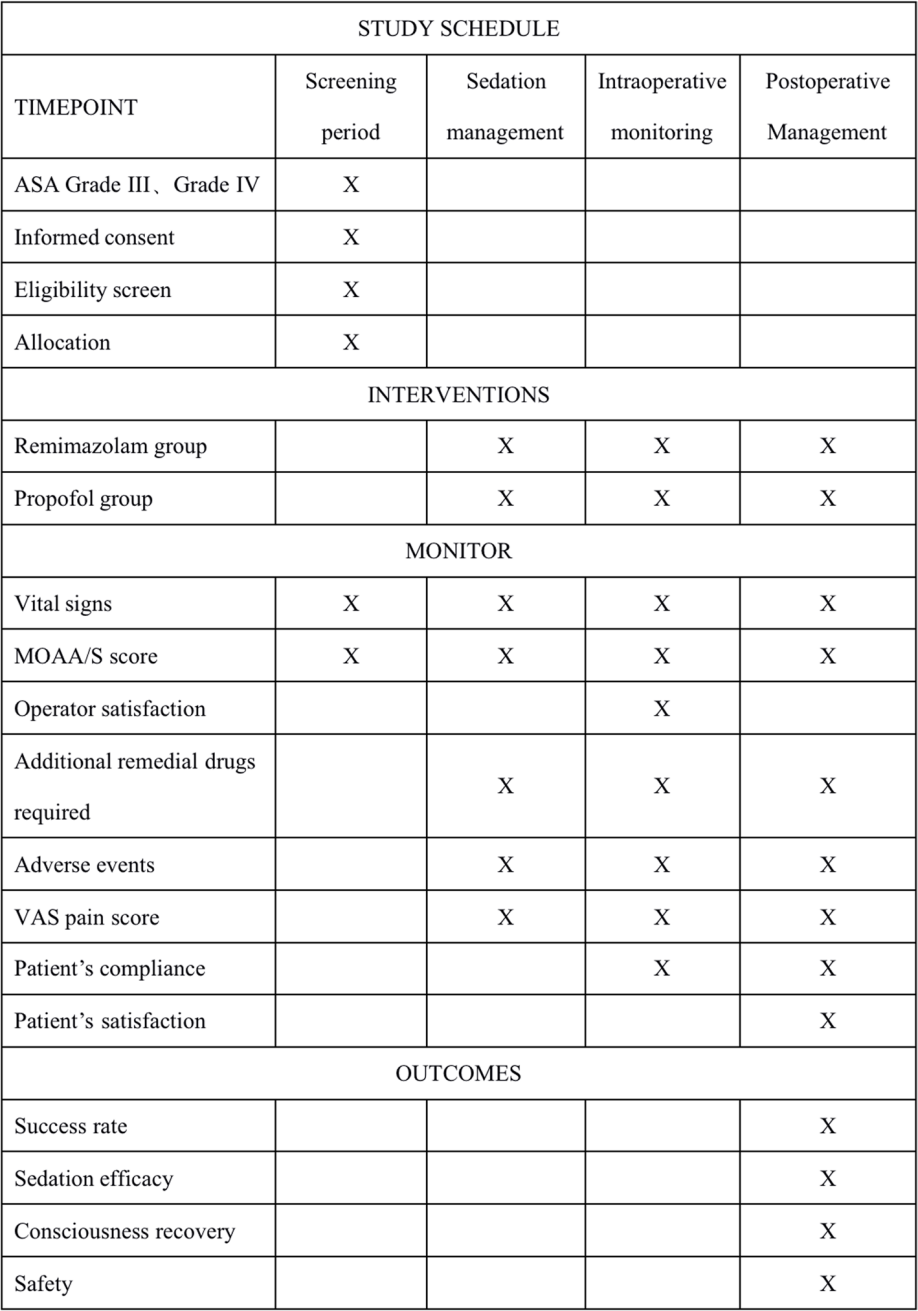
Study schedule

All the outcome measurements will take place at the baseline (before treatment) and the first day and second day during treatment (Fig. 4).

####  Primary outcome

The success rate of patients in both groups who successfully completed upper gastrointestinal endoscopy and treatment. Success will be evaluated using a composite endpoint index comprising the following criteria: (1) MOAA/S score of ≤ 3 points before endoscope insertion into the oral cavity, (2) no interruption of endoscopic examination due to inadequate sedation, (3) no occurrence of SpO_2_ < 90% during the examination, (4) no incidence of systolic blood pressure < 80 mmHg, systolic blood pressure ≥ 180 mmHg, heart rate (HR) ≤ 55 beats/minute, or *HR* > 100 beats/minute during the examination.

#### Secondary outcomes

*Sedation efficacy outcomes*: (1) MOAA/S score after single administration; (2) time required to achieve a MOAA/S score of ≤ 3 points following single administration; (3) for patients not achieving adequate sedation depth after a single dose and documentation of additional medication doses and frequency; (4) total amount and number of additional administrations of remimazolam or propofol in both groups during the entire course of upper gastrointestinal endoscopy and treatment; (5) duration from the initiation of medication injection to the completion of upper gastrointestinal endoscopy and treatment; (6) type, frequency, and dose of alternative or additional remedial drugs required when the upper limit of supplementary dosage is exceeded; and (7) operator satisfaction.

*Consciousness recovery outcomes*: (1) Time to full awakening; (2) use of sedative antagonist flumazenil, if applicable; (3) assessment of patients’ recall of upper gastrointestinal endoscopy and treatment process using the Brice questionnaire (Appendix 4) after awakening; (4) duration of patients’ recovery; and (5) overall patient satisfaction.

*Safety outcomes*: (1) Incidence of hypertension or hypotension during the procedure and in the recovery room, (2) incidence of tachycardia or bradycardia during the procedure and in the recovery room, (3) incidence of low oxygen saturation during the procedure and in the recovery room, (4) airway intervention rate during the procedure and in the recovery room, (5) incidence of coughing within 1 min after endoscope insertion, (6) incidence of coughing during upper gastrointestinal endoscopy and treatment, and (7) incidence of injection-related pain.

### Sample-size calculation {14}

The study plans to enroll 576 patients. The basis for sample size calculation is as follows: According to the literature report [7], patients undergoing upper gastrointestinal endoscopy and treatment with propofol sedation had a completion rate without sedation-related complications of 79%. Using a two-sample *z*-test for proportions to assess the primary outcome measure, the estimated non-inferiority margin is an absolute difference of 10% for the primary outcome measure. Setting a non-inferiority difference value of 0.1, a power (1-β) of 0.8, and a one-sided α of 0.025, a total sample size of 522 cases is required for the calculation. Accounting for an anticipated loss to follow-up rate of 10%, the total sample size is 576 cases (288 participants per group).

### Recruitment {15}

The principal method involves a collaborative partnership with the Endoscopy Center of the Second People’s Hospital of Futian District, Shenzhen, serving as a centralized hub for participant enrollment. Furthermore, we have employed a range of strategies, including in-person invitations during clinical visits and referrals from physicians across various medical disciplines. Designated personnel from this department will be responsible for identifying and enrolling eligible participants who meet the inclusion criteria. The confidential information of the participants will be securely stored within the Clinical Trial Public Management Platform (ResMan) and will not be disclosed to any individuals or organizations unrelated to this study.

### Statistical analysis

#### General principles{20a}

Continuous variables will be represented as means (standard deviations) or medians (minimum, maximum, or interquartile range), with the Shapiro-Wilk test applied to evaluate the normality of continuous variable distributions. Categorical variables will be expressed as frequencies (percentages).

All statistical analyses will involve two-sided tests, considering a *p*-value less than 0.05 to be statistically significant.

#### Patient recruitment and withdrawal status

The enrollment and attrition of patients will be documented.

#### Demographic and baseline characteristics

Demographic data and baseline characteristics, such as preoperative comorbidities and medication history, will be presented.

#### Efficacy evaluation{20a}

Primary endpoint evaluation: In the intention-to-treat (ITT) analysis population, a chi-square test will be used to compare the success rates of patients completing endoscopic examination and treatment between the two groups. Simultaneously, a generalized linear model will be employed to calculate the unadjusted and adjusted absolute risk differences (ARD). Adjusted variables will encompass stratification indicators (invasive procedures during upper gastrointestinal endoscopy and treatment), BMI, age, concurrent respiratory diseases, concurrent cardiovascular and cerebrovascular diseases, liver and kidney function abnormalities, and operator experience. Secondary effect sizes will be presented as odds ratios (OR) with a 95% confidence interval.

Evaluation of secondary endpoints: For the intention to treat (ITT, includes all participants who were randomly assigned, regardless of whether they completed the entire study or fully adhered to the study protocol) population, chi-square test or *t*-test will be employed for comparative analysis based on variable nature (e.g., categorical variables like low oxygen saturation, hypotension, tachycardia, intraoperative awareness, recovery delay, or continuous variables such as MOAA/S, NIBP, HR, and SpO_2_). Results will be reported as odds ratios (OR) and 95% confidence intervals using a generalized linear model. Time event variables (e.g., time to achieve a MOAA/S score of ≤ 3 points after a single dose) will be analyzed using Cox regression to describe and compare the two groups, generate Kaplan-Meier curves, express results as hazard ratios (HR), and report a 95% confidence interval.

#### Exploratory analysis {20b}

Subgroup analyses based on patients’ clinical characteristics will be performed in pre-specified post hoc exploratory analyses. Potential factors impacting the examination success rate include BMI, age, the presence of respiratory diseases, cardiovascular and cerebrovascular diseases, liver and kidney dysfunction, alcohol and drug dependence, and operator experience.

Repeated measurement data (e.g., multiple measurements of MOAA/S, NIBP, HR, and SpO_2_) will be analyzed using a generalized additive mixed model (GAMM). The fixed effect will be a stratification indicator (invasive procedures during upper gastrointestinal endoscopy and treatment), and the interaction between groups and time will be evaluated.

#### Sensitivity analysis {20c}

Broaden the primary outcome measure’s success criteria to fulfill the following two conditions: (1) MOAA/S ≤ 3 points before endoscope insertion into the oral cavity and (2) no interruption due to sedation during upper gastrointestinal endoscopy and treatment. Reassess the difference in success rates between the two patient groups. B. Utilize a per-protocol (PP, only includes participants who adhered to the study protocol and completed the entire study) analysis to select specific patients for effect estimation.

Analysis population and missing data: A multiple-imputation analysis will be performed to further evaluate whether the use of indicator variables for missing data introduces bias into our results.

#### Statistical software

All analyses were performed with the R software (version 3.4.3, R Foundation for Statistical Computing, and Empower Stats, X&Y Solutions Inc., Boston, MA, USA).

### Safety and adverse events {22}

Adverse events pose potential risks during clinical treatment. In this study, all adverse events, encompassing significant adverse events, were meticulously documented in the CRF. This documentation included the time of occurrence, clinical manifestations, treatment process, duration, outcome, and association with the drug under investigation. For patients presenting abnormal laboratory test results, follow-up measures were carried out until the results reverted to normal or pre-trial levels or until it was ascertained that the abnormality was not related to the trial intervention.

### Frequency and plans for auditing trial conduct {23}

We currently do not have a specific audit plan outlined for this study. This decision is primarily influenced by the fact that data analysis is scheduled only at the conclusion of the study, and no interim adjustments are planned in the interim period. As such, we consider an interim audit unnecessary at this stage. However, we recognize the importance of monitoring and ensuring the integrity of the study processes and will duly consider incorporating an audit plan as the study progresses.

### Plans for communicating important protocol amendments to relevant parties {25}

Any amendments to the protocol and procedures must receive prior approval from the independent Ethics Committee of the Second People’s Hospital of Futian District through the amendment process. If granted approval, the modifications will be promptly communicated and documented in the online protocol registration.

### Who will take informed consent? {26a}

Upon initiating the trial, the principal investigator will elucidate the experimental procedures and obtain written informed consent, duly signed by each participant.

### Additional consent provisions for the collection and use of participant data and biological specimens {26b}

In the consent form, participants will be requested to grant permission for the utilization of their data in accordance with local privacy policies. In the event of withdrawal, participants will be asked to indicate their willingness to permit the utilization of acquired data. It is important to note that this trial does not involve the collection and storage of biological samples.

### Confidentiality {27}

To safeguard privacy, a unique alpha-numeric identifier will be assigned to each patient to anonymize personal information and contacts.

### Data management and monitoring {19}

In this study, a paper-based data collection methodology was employed for data management. Researchers transcribed the data from the original observation records of the subjects onto a CRF, which was subsequently signed by the principal investigator and promptly submitted to the clinical trial data custodian. Data entry and management were undertaken by team members uninvolved in the research intervention, utilizing an electronic database system for data input and validation. Regular meetings were held to analyze study data and monitor the project’s progress.

### Composition of the data monitoring committee, its role, and reporting structure {21a}

In this study, we currently have not established a Data Monitoring Committee (DMC). This decision is based on the nature and scale of the trial, as well as the availability of resources. Despite the absence of a DMC, we remain committed to ensuring the scientific and ethical integrity of the trial. We will implement alternative effective monitoring measures, including regular reviews of trial progress, monitoring safety data, and ensuring the quality and accuracy of the data. Additionally, we will maintain close collaboration with regulatory authorities and the ethics committee. We are dedicated to transparently reporting and sharing the progress and results of the trial.

### Interim analyses {21b}

Analyses in this study will be limited to regular monitoring of participant numbers conducted by the study management team and will not serve as a basis for study termination. The sole predetermined criterion for terminating the study is the occurrence of data protection incidents, particularly those posing a risk of expansion. The final decision regarding study termination rests with the primary sponsor.

### Dissemination plans {31a}

The key components of our dissemination plan are outlined below: scientific journal publication, academic conferences, registration database, and patient and public communication, thereby fostering the widespread sharing of scientific knowledge and the advancement of best practices.

## Discussion

Upper gastrointestinal endoscopy is the gold standard for tumor diagnosis and treatment [8]. To provide a comfortable experience for patients and ensure their safety, medical institutions typically employ procedural sedation during upper gastrointestinal endoscopy [9]. Despite its benefits, procedural sedation presents several challenges, including timely airway emergency management, accurately controlling the depth of sedation, and managing respiratory and circulatory complications in high-risk patients [10, 11].

Procedural sedation is an anesthetic method that does not necessitate airway intervention and protection. It generally involves intravenous administration of sedatives and analgesics, with the level of sedation tailored to individual patients and endoscopic examinations or surgical procedures. Although no single drug can effectively achieve all targets, drug combination therapy is often the optimal approach for accomplishing the desired goals. Selecting appropriate sedative drugs based on their pharmacological properties is essential for ensuring safety and comfort in upper gastrointestinal endoscopic surgery [12]. Historically, benzodiazepines have been the drugs of choice for procedures sedation due to their anxiolytic and amnesic properties. Diazepam, lorazepam, and midazolam have been the primary benzodiazepines used for sedation. Midazolam, approved by the FDA since 1985 [13], remains the preferred benzodiazepine due to its rapid onset and superior amnesic effects compared to diazepam and lorazepam.

However, midazolam has significant interactions with other CYP3A4 inhibitors, such as cimetidine, erythromycin, diltiazem, verapamil, ketoconazole, and itraconazole, which can result in prolonged sedation time. Additionally, other benzodiazepine sedatives have half-lives ranging from 1.8 to 6.4 h, and their extended sedative effects may not be conducive to enhancing efficiency [2, 13].In conclusion, further research is needed to optimize drug selection and administration for procedures sedation during upper gastrointestinal endoscopy, with the aim of improving patient safety, comfort, and procedural efficiency [14].

In addition to midazolam, propofol is commonly used in procedural sedation methods due to its excellent sedative properties and short half-life. However, propofol is not without drawbacks, as it may cause aspiration pneumonia, cardiovascular and respiratory depression, hypoxia, and even necessitate tracheal intubation in some cases. Each sedative agent has its limitations for procedural sedation, and with the increasing demand for comfortable medical care, it is vital to enhance sedative quality during endoscopic examinations while ensuring safety. Developing new drugs is essential to addressing the safety and comfort concerns in procedures sedation [15, 16].

Remimazolam, a new sedative anesthetic, offers safety, comfort, and controllability advantages, presenting a new option for ideal procedures sedation. With no drug interactions with CYP3A4 inhibitors and superior pharmacokinetic and pharmacodynamic properties, such as a shorter elimination half-life, remimazolam is a promising agent for inducing and maintaining general anesthesia and sedation. It combines the characteristics of midazolam and remifentanil [17].

This study aims to compare the procedures sedative effect and safety of remimazolam with propofol in high-risk patients undergoing upper gastrointestinal endoscopy to evaluate remimazolam’s efficacy. Previous trials have predominantly included non-high-risk patients [5] or colonoscopy patients [18], making it unclear whether remimazolam provides a sedative effect and safety for upper gastrointestinal endoscopy in high-risk patients. The ASA level assessment is associated with an increased risk of adverse events or serious adverse events requiring unplanned intervention during endoscopic examinations and effectively predicts perioperative risk, serving as a valuable risk stratification tool [6]. This study seeks to apply remimazolam to procedures sedation in high-risk patients (ASA III and IV levels) undergoing upper gastrointestinal endoscopy to determine if it can provide a safe, comfortable diagnostic and treatment process and high-quality examination and surgical conditions.

This study has limitations, including the single-blind methodology, which may introduce performance bias. However, outcome evaluators and statisticians will be blinded, and efforts will be made to adjust for bias. Additionally, the study is limited to participants from a single center, and the results may only be applicable to the Chinese population. The trial’s short duration (1-day treatment) focuses solely on the short-term sedative effect and safety impact of remimazolam.

### Trial status {3}

This protocol is version 01. The participants enrollment commenced on 2022 December 17. The trial is currently in progress, with the anticipated completion date estimated to be in October 2024.

### Supplementary Information

**Additional file 1.** SPIRIT checklist.

**Additional file 2: Appendix 1.** The American Society of Anesthesiologists (ASA) Classification. **Appendix 2.** MOAA/S Scale. **Appendix 3.** Visual Analog Scale (VAS). **Appendix 4.** Brice questionnaire. **Appendix 5.** Information Leaflet for Informed Consent.

## Abbreviations

AEs: Adverse events
ARD: Absolute risk differences
BMI: Body mass index
CRF: Case report form
GAMM: Generalized additive mixed model
HR: Hazard ratios
ITT: Intention to treat
MOAA/S: Modified Observer’s Alertness/Sedation scale
NIBP: Noninvasive blood pressure
OR: Odds ratios
PP: Per protocol
SpO_2_: Pulse oxygen saturation.
