# Supplementary material for: Effect of remimazolam vs propofol in high-risk patients undergoing upper gastrointestinal endoscopy: a non-inferiority randomized controlled trial
Source: Trials. 2024 Jan 27;25:92. doi: 10.1186/s13063-024-07934-z (PMC10821577; doi:10.1186/s13063-024-07934-z)
Supplement: Supplementary file 2 — Additional file 2: Appendix 1. The American Society of Anesthesiologists (ASA) Classification. Appendix 2. MOAA/S Scale. Appendix 3. Visual Analog Scale (VAS). Appendix 4. Brice questionnaire. Appendix 5. Information Leaflet for Informed Consent. [file 13063_2024_7934_MOESM2_ESM.docx]

Appendix 1

The **American Society of Anesthesiologists (ASA)** classifies patients based on their physical status and the risks associated with surgery. This classification system was approved on October 15, 2014, and last revised on October 23, 2019. There are six ASA classification levels:

ASA Class I: Healthy, well-nourished, and with normal organ function.

Example: A healthy individual who does not smoke, drink alcohol, or drinks only in moderation.

ASA Class II: Mild systemic disease that doesn't limit normal physical activity.

Example: Smokers, social drinkers, pregnant patients, patients with mild lung disease, and patients with controlled diabetes or hypertension (mild obesity with BMI between 30 and 40).

ASA Class III: Severe systemic disease that limits daily activity but does not incapacitate the patient.

Example: Patients with poorly controlled hypertension or diabetes, chronic obstructive pulmonary disease (COPD), severe obesity (BMI ≥ 40), active hepatitis, alcohol dependence or abuse, post-cardiac pacemaker implantation, moderate decrease in cardiac output, end-stage renal disease requiring regular dialysis, history of myocardial infarction, stroke, transient ischemic attack, or coronary artery disease with coronary stent placement (more than 3 months ago).

ASA Class IV: Incapacitating systemic disease that is a constant threat to life.

Example: Recent myocardial infarction, stroke, transient ischemic attack, or coronary artery disease with coronary stent placement (within the last 3 months), severe ischemic heart disease, severe valvular heart disease, severe decrease in cardiac output, sepsis, disseminated intravascular coagulation (DIC), acute respiratory distress syndrome (ARDS), or end-stage renal disease not receiving regular dialysis.

ASA Class V: Moribund patient who is not expected to survive without the operation.

Example: Ruptured thoracic or abdominal aortic aneurysm, severe trauma, intracranial hemorrhage with mass effect, ischemic bowel in the face of severe cardiac pathology, or multiple organ/system dysfunction.

ASA Class VI: A declared brain-dead patient whose organs are being removed for transplantation.

In the classification, adding "E" signifies an emergency surgery.

Please note that this translation may vary based on context and specific medical terminology used in different regions.

Appendix 2

**MOAA/S Scale:**

0 - Unresponsive: No response to physical or verbal stimulation.

1 - Very sleep, unresponsive to mild prodding or shaking.

2 - Light sleep, easy to arouse with moderate prodding or shaking.

3 - Moderate sedation, movement or eye opening to verbal command only.

4 - Deep sedation, no response to verbal command, but movement or eye opening to physical stimulation.

5 - Light sedation, movement or eye opening to gentle shaking.

6 - Awake and alert, responsive to name-calling.

The MOAA/S scale is used to assess the level of sedation or alertness in patients. It is commonly used in anesthesia and sedation practices.

Appendix 3

**Visual Analog Scale (VAS):**

The Visual Analog Scale is a measurement tool used to assess subjective experiences or intensity of a particular parameter. It consists of a horizontal line, usually 10 centimeters in length, with two verbal descriptors anchoring each end.

Please mark on the line to indicate your level:

0 —— 1——2 ——3 ——4 ——5 ——6 ——7 ——8 —— 9——10

Descriptors commonly used for pain assessment are "No pain" at one end and "Worst pain imaginable" at the other. The patient is instructed to place a mark on the line that represents their perception or experience of the parameter being measured.

The VAS can be adapted to various subjective parameters, such as pain, anxiety, fatigue, or mood. The descriptors may vary depending on the specific parameter being assessed.

Appendix 4

**Brice questionnaire**

(1) What is the last thing you remembered before you went to sleep?

(2) What is the first thing you remembered when you woke up?

(3) Can you remember anything between these two periods?

(4) Did you dream during your operation?

(5) What was the worst thing about your operation?

Postoperatively, patients will undergo 2-3 follow-up assessments using an enhanced Brice questionnaire. A minimum of three investigators involved in the study will make determinations based on the following criteria:

1.Unawareness: No awareness and associated descriptions.

2.Dreaming: Possibly related to awareness.

3.Suspected Awareness: Unable to recall specific events related to awareness.

4.Definite Awareness: Recalled events that can be confirmed by personnel involved in the surgery.

APPENDIX 5

**Effect of Remimazolam vs Propofol in High-Risk Patients Undergoing Upper Gastrointestinal Endoscopy: A Non-Inferiority Randomized Controlled Trial**

**Information Leaflet for Informed Consent**

Dear participant,

We cordially invite you to participate in a study titled "Ramelteon Utilization in High-Risk Patients Undergoing Upper Gastrointestinal Endoscopy: A Non-Inferiority Randomized Controlled Trial." This study aims to assess the efficacy, comfort, and safety of ramelteon sedation in high-risk patients undergoing upper gastrointestinal endoscopy. The intervention involves sedation anesthesia for ASA class III and IV patients undergoing upper gastrointestinal endoscopy, using either ramelteon or propofol via intravenous injection to achieve the desired level of sedation for the completion of endoscopic treatment.

Before deciding whether to participate in this study, please take the time to thoroughly read the following information. It will help you comprehend the purpose of the study, the rationale behind conducting it, the procedures and timeline involved, as well as the potential benefits, risks, and discomforts associated with participation. If you wish, you may seek clarification from your physician or discuss this with your family and friends to aid in making an informed decision.

**Study Introduction:**

1. The purpose of this study is to investigate the success rate, comfort, and safety of using ramelteon in high-risk patients undergoing upper gastrointestinal endoscopy.

2. Currently, the standard anesthesia methods for gastrointestinal endoscopy include the use of benzodiazepines or propofol sedation, both of which are anesthetic agents administered to achieve the desired level of sedation for completing the endoscopic procedure. However, benzodiazepines provide moderate sedation depth, meeting the basic sedation requirements for clinical gastrointestinal endoscopy procedures, and can be antagonized. On the other hand, propofol induces deep sedation, leading to significant respiratory and circulatory suppression, which cannot be antagonized.

3. Description of the Treatment Method in this Study: Ramelteon, a benzodiazepine, has been approved for sedation during endoscopic examinations, with clear data demonstrating its effectiveness and safety in achieving satisfactory sedation depth during colonoscopy in high-risk patients. However, for high-risk patients undergoing upper gastrointestinal endoscopy, there is still a lack of clear clinical data regarding sedation depth and safety. This study aims to collect perioperative clinical data and compare the success rate, comfort, and safety of two standard sedation methods for high-risk patients undergoing upper gastrointestinal endoscopy.

1. The study will be divided into two groups using a randomized approach: the Ramelteon Group and the Propofol Group. In the study group, participants will receive intravenous injection of ramelteon to achieve the target sedation depth before completing the endoscopy procedure. In the control group, participants will receive intravenous injection of propofol to achieve the target sedation depth before completing the endoscopy procedure. Both groups will use fentanyl for analgesia.

5. This study will be conducted at the Gastroscopy Unit of the Second People's Hospital of Futian District, Shenzhen. It is anticipated that 576 voluntary participants will take part.

6. The primary responsible department (or team) for this study is the Department of Anesthesiology at the Second People's Hospital of Futian District, Shenzhen. This department possesses sufficient anesthesia workstations, monitoring equipment, videolaryngoscopes, tracheal intubation tools, rescue equipment, and medications to meet clinical needs, ensure safety, and support research. All anesthesiologists in this department have extensive experience from working and training at renowned tertiary hospitals within the country and hold at least intermediate-level professional titles.

7. The principal investigator of this study is Dr. Zhi Li, who is a master's degree candidate and the head of the Department of Anesthesiology. With years of study and work experience at tertiary hospitals, Dr. Li has abundant experience in anesthesia and resuscitation of critically ill patients, has been involved in municipal-level projects, and has published papers domestically and internationally.

This research has received approval from the Research Ethics Committee of the Second People's Hospital of Futian District, Shenzhen. The Research Ethics Committee of the Second People's Hospital of Futian District, Shenzhen, has reviewed this study in accordance with the principles of the Helsinki Declaration, the Civil Code, and relevant domestic medical ethics laws, regulations, and guidelines, ensuring compliance with medical ethical requirements.

**Who is eligible to participate in the study:**

1. You are eligible to participate in this study if you meet all of the following criteria: age ≥18 years; patients planning to undergo upper gastrointestinal endoscopy under sedation anesthesia; ASA class III or IV; patients who provide informed consent to participate in this study.

2. However, if you have any of the following conditions, you are not suitable for participation in this study, as these conditions could pose additional risks: patients who refuse procedural sedation; patients with a history of drug abuse and/or alcohol intoxication within the past two years; patients who have taken monoamine oxidase inhibitors or cytochrome P450 inhibitors within the past month; patients with known allergies to benzodiazepines, opioids, propofol, lidocaine, or contraindications to the use of these medications; patients who have participated in other drug clinical trials within the three months prior to the study participation; pregnant or lactating women.

Your attending physician will assess your condition and inform you whether you are eligible to participate in this study.

**What Participation in the Study Involves:**

1. Prior to your enrollment in the study, your doctor will inquire about and record your medical history and past examination results.

2. If you meet the criteria from the above examinations, the study will proceed as follows: Patients will enter the endoscopy preparation area and, 5 minutes before the examination, orally consume 10g of lidocaine gel. Upon entering the endoscopy room, patients will lie on their left side on the examination bed, with basic vital signs monitored, including non-invasive blood pressure, electrocardiogram, and pulse oxygen saturation on the right upper arm. Oxygen will be continuously administered through a nasal cannula until full recovery. An intravenous infusion of fentanyl at a dose of 0.1 μg/kg will be administered through an open vein. Patients scheduled for upper gastrointestinal endoscopy will receive intravenous injection of either ramelteon or propofol for sedation in a randomized manner: Group C will receive propofol, while Group R will receive ramelteon. The physician will assess sedation depth to determine if additional doses are required until the desired sedation level is achieved for the completion of the endoscopic examination. After the upper gastrointestinal examination is completed and the patient has recovered, the doctor will inquire about and record any changes in your condition, perform vital sign monitoring, and collect your medical history and monitoring results. The data collected will contribute to the research.

3. Other Aspects Requiring Your Cooperation:

You will need to follow the doctor's instructions regarding fasting and provide truthful responses during sedation assessment. Regarding dietary and lifestyle regulations, you should adhere to the requirements of the upper gastrointestinal endoscopy procedure.

4. Anticipated Circumstances and/or Reasons for Potential Study Termination:

Participant loss to follow-up; participant refusal for follow-up; participant or their legally authorized representative's request to withdraw from the study; investigator's judgment that the participant is no longer suitable for continued participation; inability to implement the intervention measures; failure to record trial data.

**Potential Benefits of Participation:**

Direct Benefits: Participants will receive precise sedation depth adjustment and vital sign monitoring through frequent assessments, enhancing safety during the procedure.

Indirect Benefits: This study will contribute to the development of smoother and safer procedural sedation protocols for high-risk patients undergoing upper gastrointestinal endoscopy, which can benefit patients in the future.

**Possible Adverse Reactions, Risks, Discomfort, and Inconvenience in the Study:**

The interventions employed in this study involve well-established and commonly used anesthesia methods in clinical practice and do not impose additional anesthesia-related risks on the participants. Anesthesiologists will adhere to clinical practice guidelines and employ appropriate measures based on individual patient circumstances to minimize anesthesia risks. In the event of any unforeseen circumstances, physicians will take all necessary measures to manage and rescue the situation, ensuring the utmost safety of patient anesthesia.

Intervention measures in the study can be halted based on the discretion of the attending physician, should the need arise. The time and reasons for study interruptions will be documented in the Case Report Form (CRF).

**The management of predicted adverse events in this study is outlined as follows:**

1. Laryngospasm: Mild cases: Chin lift and continuous positive pressure oxygen support until laryngospasm resolves. Moderate cases: Oxygen supplementation combined with deeper anesthesia until laryngospasm resolves. If the above measures prove ineffective, muscle relaxants may be administered to improve oxygenation or assist with tracheal intubation. This approach not only rapidly alleviates laryngospasm but also minimizes disruption to spontaneous breathing.

Possible Adverse Events, Risks, and Inconvenience:

2. Aspiration: During upper gastrointestinal endoscopic ultrasonography (EUS) examinations, a substantial amount of water irrigation may be used. This process involves positioning the head in an elevated manner to prevent water reflux. In case of reflux, immediate aspiration of oral fluid should be performed, anesthesia depth adjusted accordingly, and the procedure halted if reflux involves gastric acid or solids. Subsequent steps include direct suctioning of the oral and pharyngeal regions under visual guidance, followed by prompt tracheal intubation to ensure airway patency. Sterile saline (10-20ml) is instilled through the tracheal tube, followed by suction and oxygen administration, repeated until the aspirate turns clear and transparent to correct hypoxemia.

3. Tachycardia: Administer medication (intravenous bolus or infusion of esmolol) and/or adjust the infusion rate of sedative and analgesic drugs.

4. Bradycardia: Administer medication (intravenous atropine and/or intravenous epinephrine) and/or adjust anesthesia drug infusion rate based on depth.

5. Hypertension: Administer medication (intravenous bolus or infusion of urapidil or nitroglycerin) and/or adjust the infusion rate of sedative and analgesic drugs.

6. Hypotension: Accelerate intravenous fluid replacement and, if necessary, administer vasoactive drugs (ephedrine, phenylephrine, dopamine, or norepinephrine) and/or adjust anesthesia drug infusion rate based on depth.

7. Low SpO2: Perform mandibular advancement to open the airway and relieve posterior tongue displacement. If relief is inadequate, halt the procedure, remove the endoscope, and switch to high-flow oxygen mask ventilation. Invasive ventilation equipment like an oral pharyngeal airway, nasal pharyngeal airway, laryngeal mask airway, or endotracheal intubation may be employed when necessary.

8. Delayed Recovery: Exclude conditions such as hypoglycemia, hypotension, and hypovolemia. If necessary, administer benzodiazepine antagonist flumazenil (0.3-0.6mg intravenous injection). Physicians will take all necessary precautions to prevent and manage harm that may arise from the study.

Additionally, it should be noted that treatment outcomes can sometimes be ineffective or influenced by the presence of other conditions, leading to disease progression. This inherent treatment risk applies to all patients seeking medical care and exists even in the absence of participation in this clinical study. During the study, if treatment measures are found to be ineffective, the study will be terminated, and alternative potentially effective measures will be employed.

**Regarding Fees:**

The Second People's Hospital of Futian District, Shenzhen, will cover the cost of sedation/anesthetic medications (ramelteon/propofol) during your participation in this study for upper gastrointestinal endoscopy. However, the expenses associated with the upper gastrointestinal endoscopy procedure, treatment, and anesthesia, which are part of routine clinical care, will be your responsibility.

In the event of any harm related to the study, the Second People's Hospital of Futian District, Shenzhen, will cover your medical expenses and provide appropriate financial compensation as per legal and regulatory requirements.

If you require treatment and examinations for concurrent medical conditions, these will not be covered under the scope of the study and will not be provided free of charge.

**Is Personal Information Kept Confidential?**

Your medical records (research case report forms, laboratory reports, etc.) will be securely stored within the hospital, and monitoring examination results will be documented in your outpatient medical records. The researcher, sponsor representatives (if applicable), and the ethics committee will be permitted to access your medical records. Any public reporting of study results will not disclose your personal identity. We will make every effort to safeguard the privacy of your personal identity and information within the limits allowed by law.

**Seeking More Information?**

You are welcome to raise any questions you may have about this study at any time. Your doctor or researcher will provide you with their contact number to address your inquiries.

If you have any complaints related to your participation in the study, please contact the Ethics Office of the Second People's Hospital of Futian District, Shenzhen (Phone: 0755-83150785).

Should any significant new information arise during the course of the study that might impact your willingness to continue participating, your doctor will promptly inform you.

**Voluntary Participation and Withdrawal from the Study:**

Your participation in this study is entirely voluntary. You have the right to decline participation in this study or to withdraw from it at any point without affecting your relationship with your doctor. Such decisions will not result in any loss of other medical benefits for you.

In the best interest of your well-being, your doctor or researcher may also terminate your participation in the study if deemed necessary.

Should you choose not to participate in this study or decide to withdraw during its course, there are numerous alternative sedation methods available, such as intravenous midazolam, etomidate, and other sedative agents. You are not obligated to join this study solely for the purpose of sedation during your examination.

If you withdraw from the study for any reason, you may be asked about your experience with the study procedures. If deemed necessary by the doctor, you may be requested to undergo laboratory tests and physical examinations. These measures are designed to safeguard your health.

**What Should You Do?**

Before making a decision to participate in the study, please make every effort to inquire with your doctor about any questions you may have until you have a complete understanding of this research.

The decision to participate in this study is entirely yours. You are encouraged to discuss it with your family or friends before making a decision.

Thank you for reviewing the provided materials. If you decide to participate in this study, please inform your doctor or research assistant, who will help coordinate all study-related matters for you.

Please keep this information for your reference.

**Signature Leaflet for Informed Consent**

Project Title: Effect of Remimazolam vs Propofol in High-Risk Patients Undergoing Upper Gastrointestinal Endoscopy: A Non-Inferiority Randomized Controlled Trial

Sponsor/Issuing Institution: The Second People's Hospital of Futian District, Shenzhen

Ethics Review Approval Number: Clinical Research Ethics Committee of The Second People's Hospital of Futian District, Shenzhen, Approval No. 2022-06

**Consent Statement:**

I have read the aforementioned introduction regarding this study and have had the opportunity to discuss and raise questions about this study with my doctor. All questions I have raised have been satisfactorily answered.

I am aware of the risks and benefits associated with participating in this study. I understand that my participation is voluntary and confirm that I have had sufficient time to consider it, and I am aware that:

● I am free to consult my doctor for more information at any time.

● I have the right to withdraw from this study at any time without facing discrimination or retaliation. My medical treatment and rights will not be compromised.

Furthermore, I understand that if I choose to withdraw from the study, especially due to reasons related to the medication, it would be highly advantageous for both myself and the entire study if I inform my doctor about any changes in my medical condition. This would involve undergoing appropriate physical and laboratory examinations.

In the event that I require any other form of medication for my illness, I agree to seek my doctor's advice beforehand or inform my doctor truthfully afterward.

I consent to the inspection of my study records by regulatory authorities, ethics committees, or representatives of the sponsor.

I will receive a signed and dated copy of the informed consent form.

Lastly, I have made the decision to participate in this study.

Participant's Signature: ____________

Date: ____ Year ____ Month ____ Day Contact Number: ________________

Guardian/Authorized Representative's Signature:___________

Relationship to Participant: __________(Note: If the participant lacks capacity or has limited capacity to provide consent, the informed consent may be signed by their guardian or authorized representative.)

Date: ____ Year ____ Month ____ Day Contact Number: ________________

I confirm that I have explained the details of this study, including the rights, potential benefits, and risks, to the participant and provided them with a signed copy of the informed consent form.

Investigator Signature: ____________

Date: ____ Year ____ Month ____ Day Contact Number: ________________
